# *MuscleX-DI*: an integrated data analysis package for X-ray scanning diffraction imaging experiments

**DOI:** 10.1107/S1600577525010859

**Published:** 2026-01-01

**Authors:** Rama S. Madhurapantula, Jiranun Jiratrakanvong, Grant Nikseresht, Nikhil Miskin, Ran Huo, Jules Nabon, Thomas C. Irving, Gady Agam, Joseph Orgel

**Affiliations:** a Department of Biology Illinois Institute of Technology Chicago IL60616 USA; b Center for Synchrotron Radiation Research and Instrumentation (CSRRI) Illinois Institute of Technology Chicago IL60616 USA; c Department of Computer Science Illinois Institute of Technology Chicago IL60616 USA; d Illinois Institute of Technology Chicago IL60616 USA; e Biophysics Collaborative Access Team (BioCAT) Illinois Institute of Technology Chicago IL60616 USA; f Department of Biomedical Engineering Illinois Institute of Technology Chicago IL60616 USA; Tohoku University, Japan

**Keywords:** X-ray diffraction, scanning diffraction imaging, data analysis, Python, synchrotron radiation

## Abstract

X-ray scanning diffraction can be used to raster-scan tissues to elucidate structural organization and other physical properties. A comprehensive data analysis package targeted to take raster X-ray diffraction (XRD) scans is vital in integrating data at multiple length and resolution scales. *MuscleX-DI* is an open-source software suite designed for the analysis and visualization of scanning XRD data. Developed in Python and compatible with multiple operating systems, *MuscleX-DI* provides an end-to-end pipeline for processing diffraction patterns and generating 2D visualizations of calculated measurements. The software supports both a graphical user interface and a command-line interface, offering tools for calibration, masking, azimuthal and radial integration, and heatmap generation. By streamlining the analysis of large XRD datasets, *MuscleX-DI* facilitates the extraction of structural information from heterogeneous samples, enabling new insights into tissue architecture and biomolecular organization. The scanning diffraction methodology can be applied to study complex tissues and assemblies at various length scales. Example applications include characterizing myelin organization in brain tissue and observing improved drug availability in tumors when used with ECM-degrading enzymes.

## Abstract 2

*MuscleX-Diffraction Imaging* (*MuscleX-DI*) is an integrated, open-source software suite for data reduction and visualization of scanning X-ray diffraction (XRD) experiments. It is developed in Python and runs on Linux, Microsoft Windows or macOS. *MuscleX-DI* offers an end-to-end software analysis pipeline that ingests diffraction patterns and raster scan parameters to generate 2D visualizations of various calculated measurements from raw XRD patterns. Modules can be run via a graphical user interface or in a ‘headless mode’ from the command line. We provide an overview of the general structure of the *MuscleX-DI* software package.

## 1. Introduction

Annotation of structural and functional details of various tissues and tissue complexes is of great value to the scientific community. Multi-modal and multi-scale methodologies that elucidate material and mechanical properties are proving to be important tools in biomedical innovations. With the advent of fast imaging hardware and software, multi-modal imaging has become one of the most common methodologies in studying native and diseased biological tissues at various levels (Schulz *et al.*, 2012 ▸; Barlow *et al.*, 2020 ▸). It is important, however, to study tissue structures in their native or near-native state to understand how disease affects these structures. One difficulty is that most high-resolution microscopy techniques often require sample preparation and derivatization steps that alter the native structure of the tissues. This is particularly problematic when the changes brought about by disease or deformation of tissues are on the same scale as those introduced by the sample treatments. For instance, fixation of brain tissues using glutaraldehyde is commonly used with autofluorescence microscopy. In this case, the fixation itself causes myelin packing to shrink by over 25%, while evidence suggests that mild traumatic brain injury (TBI) causes a change in packing that is significantly smaller than that (Orgel *et al.*, 2019 ▸).

X-ray diffraction (XRD) has long been used to study molecular packing structures of fibrous tissues (Orgel *et al.*, 2019 ▸; Orgel *et al.*, 2014 ▸; Irving *et al.*, 2011 ▸; Orgel & Irving, 2006 ▸; Wess & Orgel, 2000 ▸). This method has led to unprecedented molecular insights into changes in local and long-range packing structure through the application of mechanical forces and chemical treatments (Madhurapantula *et al.*, 2020 ▸; Orgel *et al.*, 2019 ▸; Madhurapantula *et al.*, 2017 ▸). While X-ray fiber diffraction studies on isolated, homogeneous, samples have been very fruitful, advances in synchrotron X-ray technologies, including increasingly smaller source sizes allowing microbeams containing high X-ray flux, and time framing X-ray area detectors (25–1000 Hz), provide the ability to quickly and efficiently interrogate micrometre scale regions of the heterogeneous samples, providing new scientific opportunities.

### 1.1. Scanning diffraction imaging

Scanning diffraction imaging is an experimental methodology that leverages the intrinsic power of X-ray diffraction to elucidate molecular packing structures *in situ* within the context of tissue samples. By employing a microbeam (typically 2–50 µm) and raster scanning the sample, diffraction scanning allows researchers to map the distribution of specific components, such as collagen, myelin and amyloids, within the tissue (Liu & Makowski, 2022 ▸; Karunaratne *et al.*, 2013 ▸). The packing of these semi-crystalline diffracting tissue elements produces diffraction patterns with orientation and intensity characteristics which can be useful in identifying changes to these packing structures in disease. The orientation of these fibrillar or lamellar packing structures in relation to the tissue hierarchy and organization can also be determined using these diffraction patterns, thereby providing a nanoscopic view of the packing within the context of the meso-scale (millimetre to centimetre level) tissue or organ. Information on molecular packing and distribution of strongly diffracting materials can be of great use to develop high-resolution multi-scale models of tissues. The diffraction patterns collected using this experimental capability opens new avenues for understanding the heterogeneity of biological tissues and the relationships between their structure and function. When combined with traditional microscopy methods, these data can also be used to study long-range structural changes and their correlations to functional deficits.

The diffracting materials at a given position in the sample will have varying degrees of orientation so that meridional reflections will be spread out into arcs whose angular width depends on the degree of orientation. In the isotropic case, these reflections form complete rings. The information that is desired to extract from these images is:

(i) The total amount of diffraction material which will be proportional to the integrated intensity.

(ii) The chemical identity of the diffracting material which can be determined from its *d*-spacing.

(iii) The orientation of the long axis of the diffracting material (in which direction the molecules are ‘pointing’).

(iv) The degree of disorientation around this axis, determined by the angular width of the meridional arcs.

This paper provides a comprehensive overview of the ‘Scanning Diffraction’ module in *MuscleX*, detailing its functionalities, and illustrating applications in various research contexts.

## 2. Related software

To appreciate the uniqueness of *MuscleX-DI*, it is important to place it in existing software for scanning X-ray diffraction (XRD) and fiber-diffraction analysis. Several established packages offer overlapping capabilities for detector calibration, integration and mapping. These include: *pyFAI*, *DAWN*, *Fit2D*, *DPDAK*, *XRDUA*, *GSAS-II*, *Dioptas*, and *HEXRDGUI*. Each of these targets has a different level of the data analysis workflow, from real-time reduction to quantitative refinement.

### 2.1. Overview of *MuscleX-DI*

*MuscleX-DiffractionImaging* (*MuscleX-DI*) is specifically designed for scanning diffraction of oriented or partially oriented materials such as muscle, collagen, and amyloid. It automatically detects diffraction rings, performs azimuthal integration, and calculates for each reflection the orientation angle and angular spread (σ). The software outputs maps of total intensity, angular range, orientation vectors, and ellipticity across raster scans. Both interactive and command-line (headless) modes enable high-throughput analysis of large datasets. Internally, *MuscleX-DI* employs *pyFAI* for calibration and integration but extends it with fiber-specific ring detection and orientation mapping functionality (Jiratrakanvong *et al.*, 2024 ▸; BioCAT-APS, 2024 ▸).

### 2.2. Comparison with related tools

*pyFAI* (Kieffer *et al.*, 2023 ▸) is a robust Python library for fast azimuthal integration and diffraction geometry calibration. Its diff_map module and FiberIntegrator API allow mapping and GIWAXS/fiber geometries, respectively. However, users typically script custom analysis to derive per-ring orientation and angular-dispersion maps comparable with those produced directly by *MuscleX-DI*.

*DAWN* (Basham *et al.*, 2015 ▸; Filik *et al.*, 2017 ▸) (Diamond Light Source) provides a full-featured GUI for synchrotron data processing. Its Mapping Perspective supports multi-modal visualization and automated workflows for SAXS/WAXS/PXRD, but lacks direct per-ring fiber-orientation analysis.

*Fit2D* (Hammersley, 2016*a* ▸), once a standard at synchrotron facilities, offers calibration, masking, and caking through an X11 GUI. Though capable of reciprocal-space mapping, it is now largely legacy software without explicit support for fiber-diffraction orientation metrics.

*DPDAK* (Benecke *et al.*, 2014 ▸) offers a plugin-based GUI for rapid SAXS/WAXS analysis and live scan processing, built atop *pyFAI*. Its modular design supports user-defined analysis pipelines, though fiber-specific metrics require customization.

*XRDUA* (De Nolf & Janssens, 2010 ▸) focuses on micro-XRPD and XRPD tomography, automating calibration and applying Pawley or Rietveld refinement to generate quantitative phase maps. It complements *MuscleX-DI* by addressing the structural modeling stage rather than orientation mapping.

*GSAS-II* (Toby & Von Dreele, 2013 ▸) provides comprehensive tools for Rietveld refinement, texture, and strain analysis from 2D detector data. It is typically used downstream of *MuscleX-DI*, after integration.

*Dioptas* (Prescher & Prakapenka, 2015 ▸) is a lightweight GUI for rapid calibration, masking, and batch integration of area-detector data, widely adopted in high-pressure and powder diffraction experiments.

*HEXRDGUI* (Sharma *et al.*, 2023 ▸) serves high-energy diffraction microscopy (HEDM/3DXRD) workflows, focusing on polycrystalline grain mapping rather than fiber-like scattering.

### 2.3. Summary

In contrast to the above-mentioned tools, *MuscleX-DI* provides an integrated workflow combining ring detection (via the Log-Central-Difference and Multiple Conical Integration methods), orientation-angle extraction, and direct generation of spatial heatmaps in a single application. Other packages such as *pyFAI*, *DAWN*, and *DPDAK* can achieve similar steps only through custom pipelines or external scripting. *MuscleX-DI* therefore serves as a bridge between beamline-level data reduction and biological interpretation, emphasizing usability and reproducibility in scanning diffraction imaging of soft and fibrous tissues.

A comparison of *MuscleX-DI* with related diffraction-analysis tools if provided in Table 1 ▸.

## 3. MuscleX-DI

A scanning diffraction imaging experiment produces large volumes of data in the form of a series of diffraction patterns. Analyzing these patterns requires a significant amount of computational power but more importantly a suitable software package that can analyze these data. An ideal software package for this methodology would accomplish the following objectives:

(i) Be easy to install and operate, preferably with a graphical user interface (GUI) and that can be deployed on multiple operating systems (UNIX, Linux, Windows and MacOS).

(ii) Be able to ingest scan parameters in human-readable formats, *i.e.* start and end positions of scan, and use them to calculate and present a 2D representation of data from diffraction patterns.

(iii) Ability to export data to formats that can be used for analyses using other programs.

(iv) Do all the above-mentioned tasks with minimal human intervention.

The scanning diffraction imaging module of *MuscleX*,‘*MuscleX-DI*’, is conceived as a comprehensive set of tools for analyzing and visualizing diffraction data obtained from scanning diffraction imaging experiments. *MuscleX* is an open-source software suite designed for analyzing X-ray fiber diffraction patterns, with a particular focus on striated muscle tissues (Jiratrakanvong *et al.*, 2024 ▸). It offers a comprehensive toolkit for data reduction and analysis, enabling researchers to extract meaningful structural information from complex diffraction patterns. *MuscleX* is written in Python and is compatible with various operating systems, making it accessible to a wide range of users. Its user-friendly interface and efficient processing capabilities make it a valuable tool for researchers in the field of biophysics and structural biology. *MuscleX-DI* utilizes several general features of the overall *MuscleX* package such as file input background subtraction, calibration, curve fitting and plotting (Jiratrakanvong *et al.*, 2024 ▸) but with specific adaptations for scanning diffraction imaging.

Several other software packages can accomplish these data analysis and visualization targets with differing levels of capabilities and ease of use (Basham *et al.*, 2015 ▸; Prescher & Prakapenka, 2015 ▸; Benecke *et al.*, 2014 ▸; Bian *et al.*, 2006 ▸; Rajkumar *et al.*, 2007 ▸; Hammersley, 2016*b* ▸; Ashiotis *et al.*, 2015 ▸; Rodriguez-Navarro, 2006 ▸). Many of them perform each step of analysis separately and require the user to build a ‘pipeline’ and perform further intermediate steps that often require developing additional scripts or programs to format data for input into the next step. Table 1 ▸ lists some existing toolkits. *MuscleX-DI* also delivers a ‘one-stop’ solution by providing end-to-end analyses and visualization modules. The routine can input image data types supported by the ‘FabIO’ library (Bergbäck Knudsen *et al.*, 2013 ▸) covering most commonly used, commercially available X-ray detectors, with tiff and hdf5 files being the most extensively tested by us (Jiratrakanvong *et al.*, 2024 ▸; Lee *et al.*, 2019 ▸). The *pyFAI* package developed at the ESRF is used for accelerated radial integration, image calibration and masking (Ashiotis *et al.*, 2015 ▸).

## 4. Modules and functionality

### 4.1. Overview

**Figure 1:**
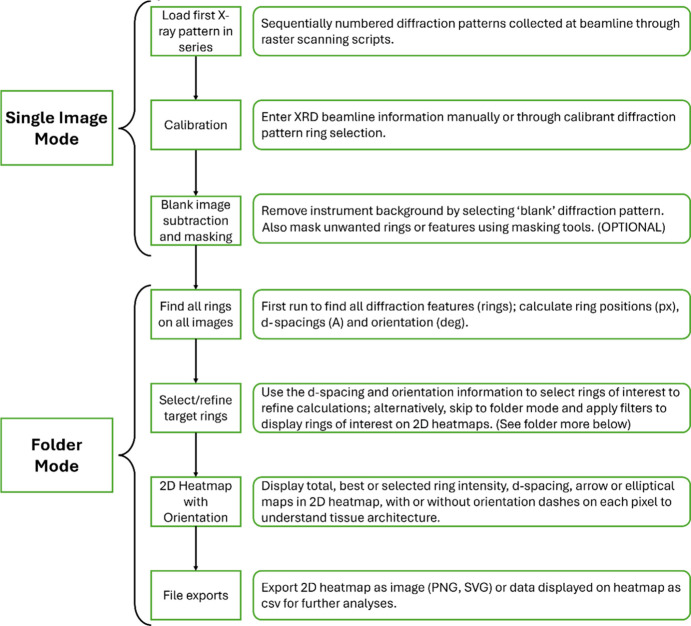
General flow of operations in *MuscleX-DI*. The rectangles on the left indicate individual workflow steps and a description and/or purpose of that step is described in the rectangle next to it. All analyses begin in ‘Single Image’ mode. By default, all rings are identified and displayed on the image that is loaded on into *MuscleX-DI*. The instrument information (sample-to-detector distance, X-ray wavelength, detector type) can be applied by selecting a calibrant diffraction pattern (lanthanum borate, silver behenate *etc*.) and choosing the ring that denotes the principal repeat, or by manually entering these parameters. A blank image (typically a diffraction pattern from the matrix in which the sample section is embedded within the sample holder) can be used to subtract the ‘instrumental’ background. Masks can be applied to exclude specific areas of images, particularly around the beamstop. All diffraction features (rings) are identified by default and users can then select target rings to analyze across datapoints through selection on the user interface. The complete diffraction sequence is then analyzed in ‘Folder Mode’. Total ring intensity, *d*-spacing of each ring and fiber alignment are calculated for each diffraction pattern in the sequence and are written to output files (see sections below). The 2D heatmap interface is then used to display a stitched map where each pixel can denote one of the parameters that was calculated. Fiber orientation can be toggled on or off using the respective buttons. The heatmap can then be exported in various formats for further analyses and representation.

A high-level overview of the flow of operations to analyze a scanning diffraction dataset is presented in Fig. 1 ▸. More specific details on operations and user manuals for using *MuscleX-DI* are available in the online documentation (Lee *et al.*, 2019 ▸).

### 4.2. Single image and folder modes

**Figure 2:**
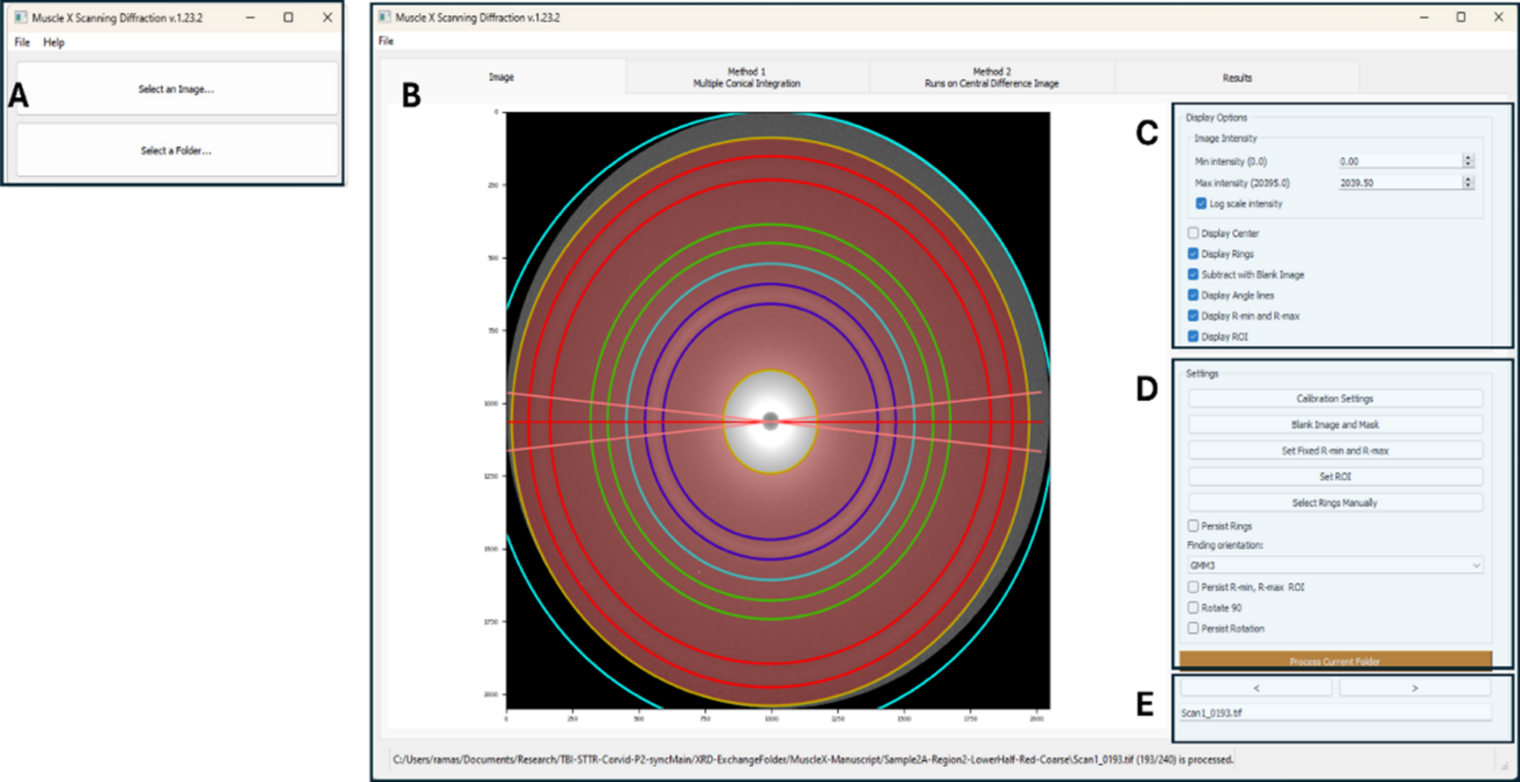
*MuscleX-DI* in ‘Single Image’ mode. Panel A shows the interface used to select single image or folder mode. Users start most analyses in the single image mode by clicking ‘Select an Image…’. This will load the images into the interface shown in Panel B. Panel C shows display options to scale intensities displayed on the diffraction pattern. The ‘Settings’ in panel D can be used to update integration, blank image and masks, inner and outer radial distance used for analyses, regions of interest in this range, or the option to select specific rings across the dataset. After selecting the appropriate settings, users can process the entire folder with sequentially numbered diffraction patterns from a scan to apply the same settings for the dataset.

All analyses begin in the ‘Single Image’ mode (see Fig. 2 ▸). In this mode, the user is first presented with a screen for calibration selection (see the following subsection on calibration below for further details[Sec sec4.3]). Once an image is loaded, users can open any one image within the series using the file number sequence and progress buttons as seen in Fig. 2 ▸(*e*). The goal is (i) to find a diffraction pattern with all target diffraction features present, usually in the form of full or partial rings, (ii) to allow the user to specify regions and rings of interest in the diffraction pattern via a graphical interface that can be applied to the full sequence of diffraction patterns. By default, all the rings present in a diffraction pattern will be detected and analyzed. Each ring (diffraction peak) is depicted by two color-coordinated concentric circles, the inner denoting the beginning of the diffraction peak in the radial direction and the outer ring denoting the outer radial extent of the peak. A principal axis of diffracting peaks is also displayed as a red line with two more lines denoting the spread of the diffraction peak around its center.

### 4.3. Calibration

*MuscleX* offers in-built calibration settings for a range of commonly used detectors and chemical and biological calibrants. It also provides an interface for users to manually enter the sample to detector distance (S_dd_), detector pixel size (or select from a range of detectors), and X-ray wavelength (Lambda).

**Figure 3:**
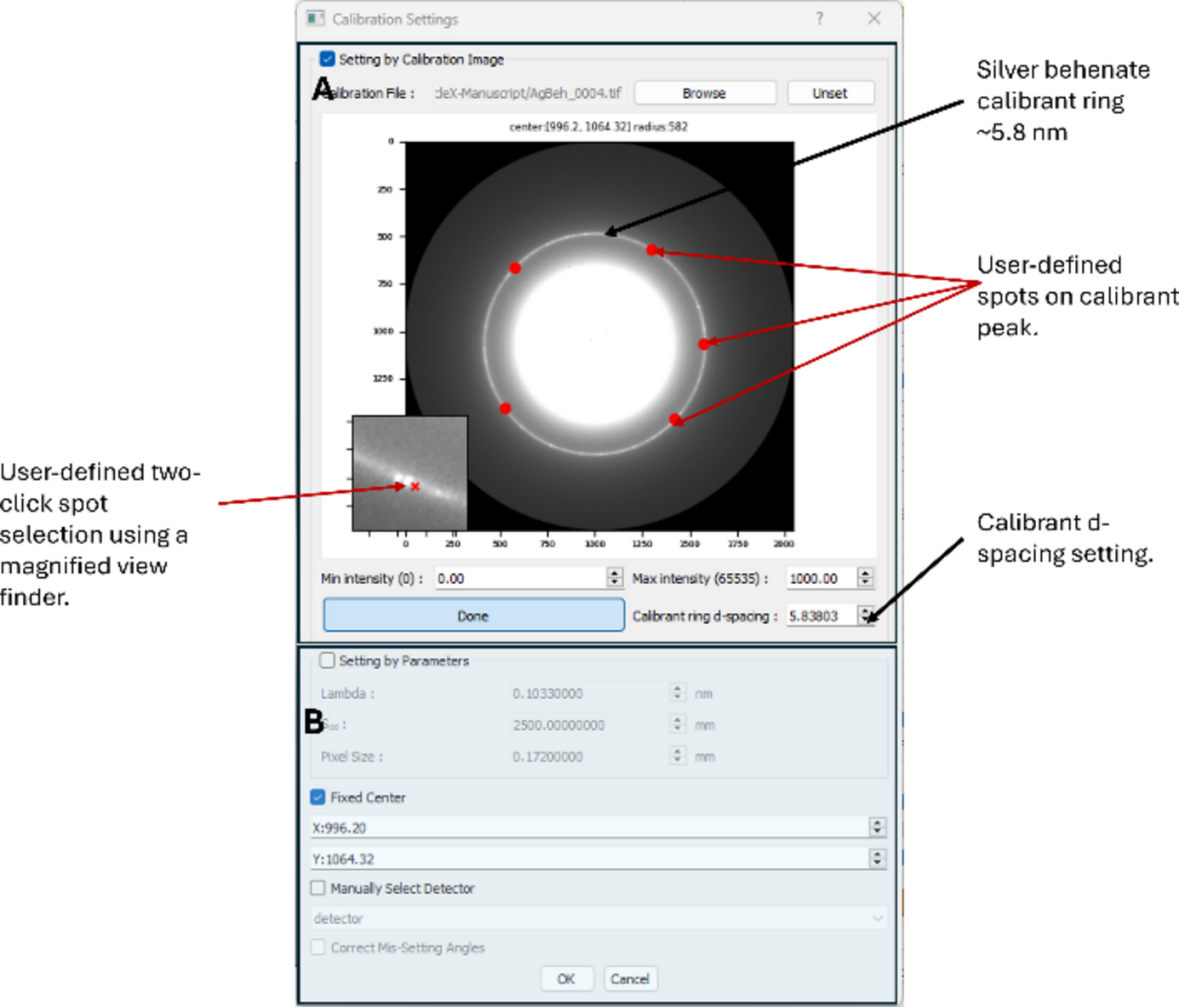
The calibration interface on *MuscleX-DI*. The module uses *pyFAI* to start with default settings and silver behenate as a default calibrant. Upon loading the diffraction pattern from the calibrant (silver behenate in this case), the user is prompted to select at least five spots on the calibrant’s first order diffraction peak (*i.e.* the reflection that marks the principal repeat). The program will then fit a new ring, and calculate the sample-to-detector distance. A detector is selected manually (defaults to the mar165 CCD). Additionally, the interface also allows users to enter these parameters manually, as shown in Panel B of the figure.

A key feature here is the two-click method to define points on the calibrant diffraction peak which is inspired by a similar design within *Fit2D* (Hammersley, 2016*b* ▸). This allows accurately defining the diffraction peak and leads to precision when calculating *d*-spacings of the various diffraction peaks in samples. Masking may be performed for an image series using the built in *pyFAI* masking tools. See Fig. 3 ▸.

### 4.4. Fast azimuthal integration using *pyFAI* – data and outputs

All diffraction patterns are analyzed to find rings and diffraction pattern orientation using the *pyFAI* package. Azimuthal integration involves summing the intensities along a circular path at a constant radius. This process effectively averages the signal around a specific scattering angle (2θ) in the detector plane. This integration method converts a 2D diffraction image into a 1D profile, representing the intensity as a function of the scattering angle. Peaks or rings are identified on the series of diffraction images via two main methods, *i.e.* Multiple Conical Integration and the Log Central Difference Method. The outputs from these methods are used to ‘merge’ rings to present higher accuracy during detection.

#### 4.4.1. Multiple conical integration

**Figure 4:**
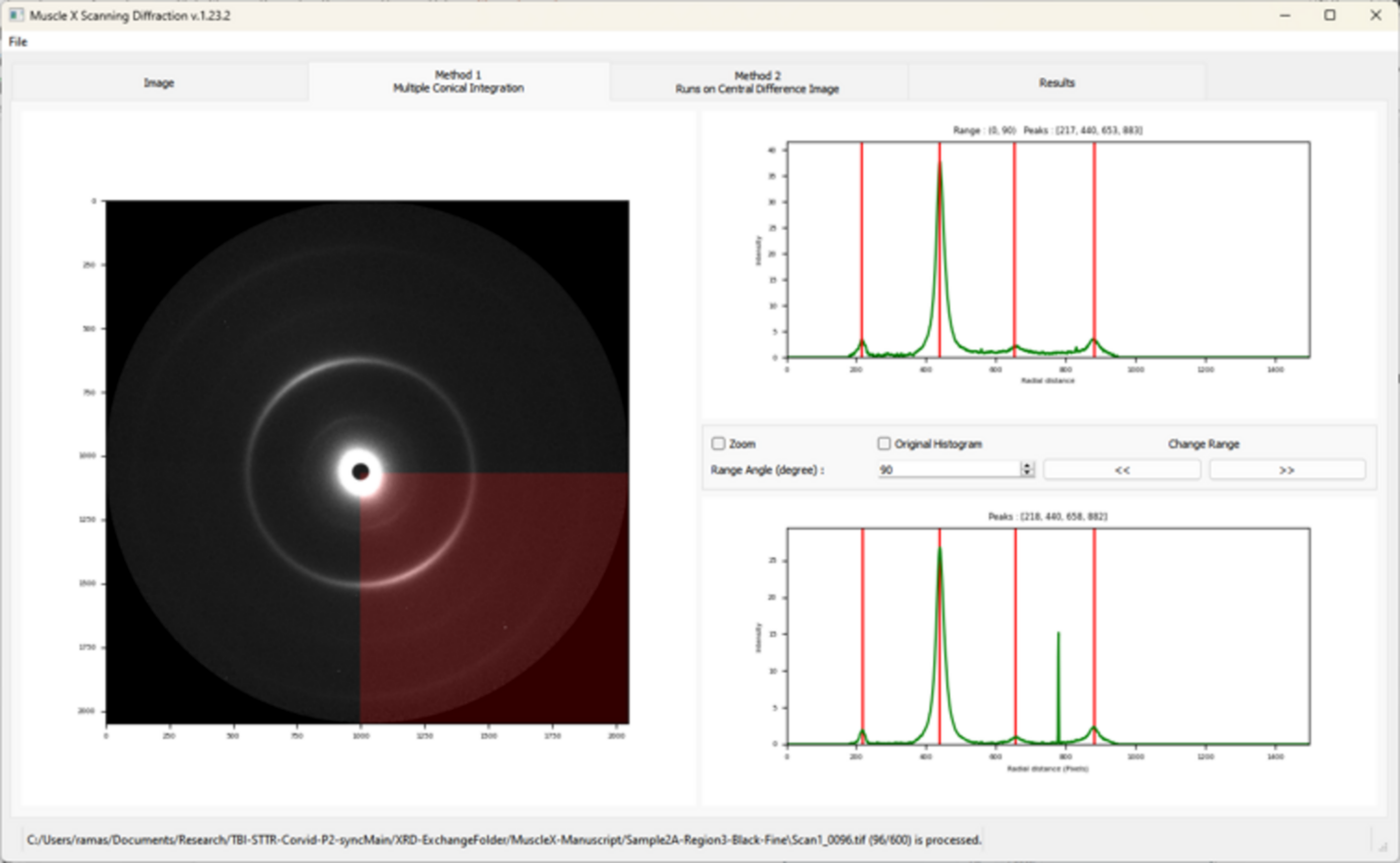
Multiple Conical Integration mode with a default angular range of 90°. The centers of peaks in the diffraction pattern are displayed as the red lines on the 1D profile. Users can change the range angle and view the next range using the navigation buttons between the 1D plots.

In this method, the *pyFAI* library is utilized to calculate the 1D integrated intensity within a pie-shaped region centered on the diffraction pattern (Ashiotis *et al.*, 2015 ▸). The angular range of this region is user-definable, with a default value of 90°. Multiple 1D integrated intensity traces, one for each angular range, are generated. For each angular range, the program identifies peak locations within the corresponding integrated intensity trace. A peak is considered to represent a ring if it appears in at least one-quarter of the total number of angular ranges. For instance, with eight angular ranges, a peak present in at least two ranges is classified as a ring. See Fig. 4 ▸.

#### 4.4.2. Central-difference based ring detection

This method proves particularly useful in identifying weak diffraction peaks that may be obscured by the presence of stronger peaks within the pattern. Let *I*(θ, *r*) denote the 2D azimuthally integrated intensity as a function of azimuthal angle θ and radius *r* after background removal. To enhance ring-like structures, which are localized and narrow along the radial direction, we apply a discrete central-difference operator along *r*.

We form a central-difference-like image by combining three radially shifted copies of *I*(θ, *r*) with a stencil (−1, 2, −1),
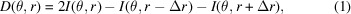
where Δ*r* = *q*_0_ is a fixed radial offset measured in pixels (in the current implementation *q*_0_ = 10). This expression is (up to a constant factor) the negative second finite difference along the radius and thus approximates the negative second radial derivative of *I*. It yields large positive values at radii where the intensity profile is sharply peaked (*i.e.* at diffraction rings) and small values in slowly varying regions.

**Figure 5:**
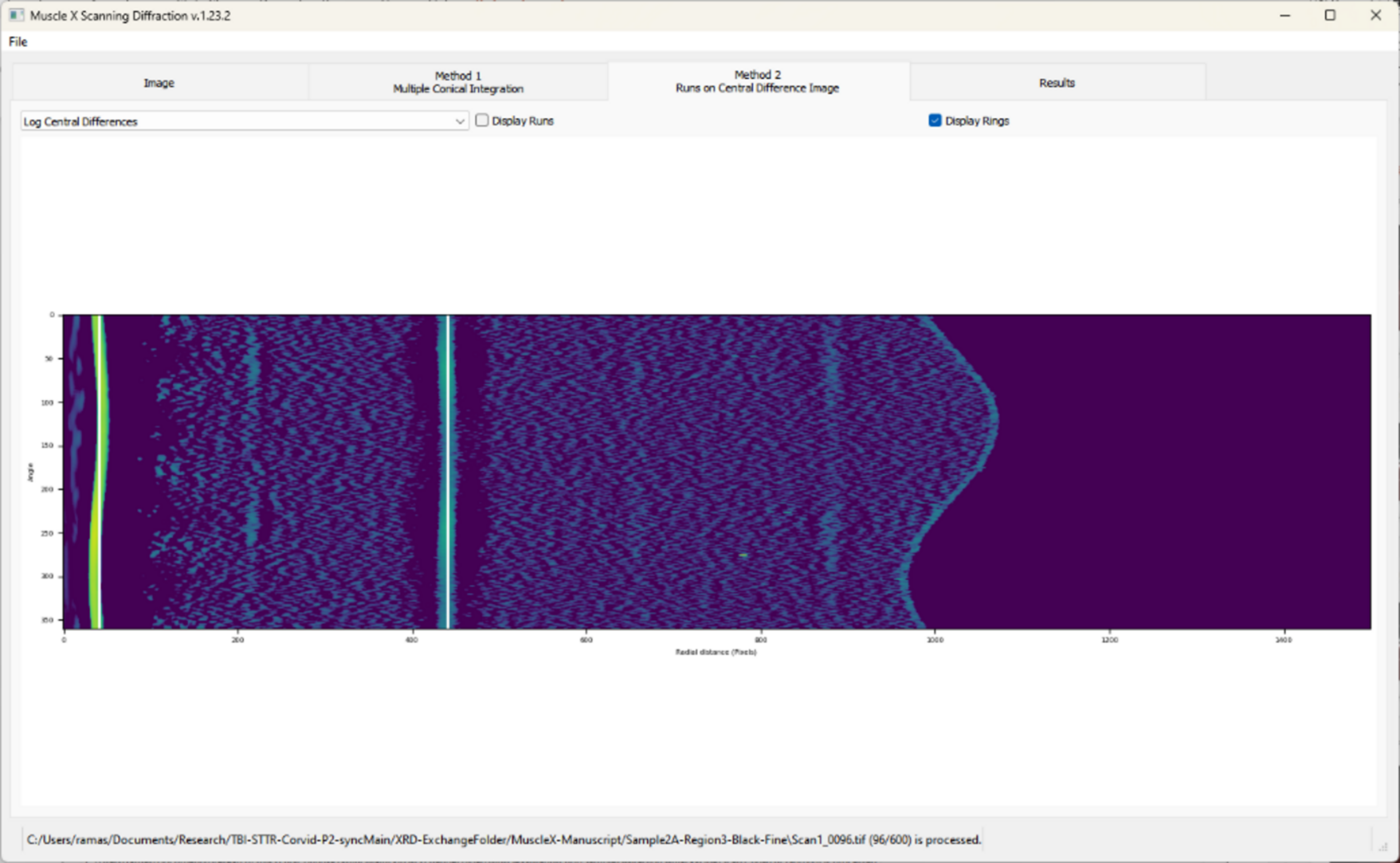
The log central differences, *L*(θ, *r*), displaying peaks in white. The runs can be displayed by toggling them on and off using the checkbox. Users also have the option of displaying the 2D integration profile, central differences, and Log central differences using the dropdown menu.

To avoid numerical issues and to compress the dynamic range, we clip negative values of *D* to zero and add a small constant ɛ,
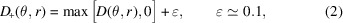
and we define the log central differences image as 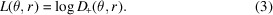
Fig. 5 ▸ displays this log-transformed image *L*(θ, *r*), where ring locations appear as bright arcs. We then scan each column (fixed *r*) of *L*(θ, *r*) to identify long contiguous ‘runs’ of high intensity, which correspond to segments of diffraction rings. These runs are subsequently grouped into rings based on continuity across the angular direction and used as input for the subsequent ring merging and modeling steps.

#### 4.4.3. Ring merging

The data from the two methods of peak/ring finding are then used to ‘merge’ rings to their best position. This two-method approach allows the detection of weaker diffraction data in the presence of stronger diffraction peaks. Rings with similar distances are averaged. For instance, if the conical integration method reported rings at 50, 80, 118 pixels and the log central differences method reported rings at 82, 120, 180 pixels, the final rings will be at 50, 81, 119, 180 pixels.

### 4.5. Determining fiber alignment from diffraction patterns

**Figure 6:**
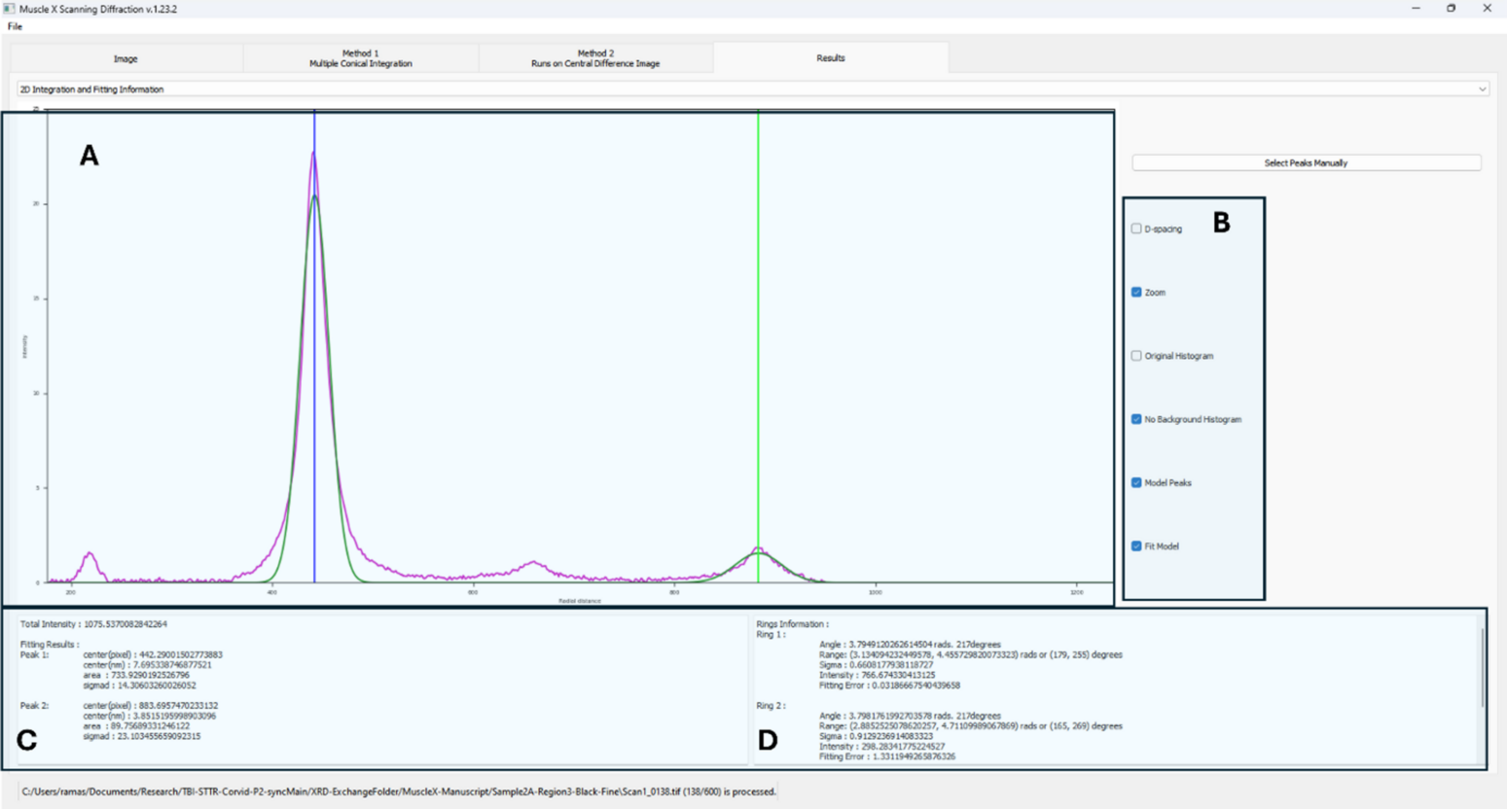
Results window of the single image integration interface in *MuscleX-DI*. Panel A displays the 1D azimuthal integration profile and the model Gaussian peaks that are fit on each of the diffraction peaks. The dropdown menu at the top is used to toggle between displaying azimuthal and radial integration profiles. Panel B is used to tweak the various display settings on this interface. The D-spacing option will display a reciprocal space map (X-axis is changed to nanometres) showing the orders of diffraction. Users can select peaks from the 1D plot as well for higher accuracy. Panels C displays the results of azimuthal integration and ring merging. This includes the center of the peak [in pixels and nm (*d*-spacing)], area of the curve, and standard deviation of the peak fit calculated from the FWHM of the model peak. Panel D displays the results of radial integration and curve fitting. This shows the diffraction orientation (radians and degrees) angular range of the peak (start and end of the diffraction peak from background), intensity of the ring (peak height) and fitting error. These parameters are also available in the ‘rings.csv’ file.

Radial integration sums the intensities along a radial line from the center of the diffraction pattern at a fixed azimuthal angle. This extracts information concerning the intensity variation along a specific radial path within the diffraction pattern. For fibrillar assemblies (*e.g.* collagen, muscle), the direction of the axis is similar or identical to the axis of the principal diffraction. For lamellar assemblies (myelinated nerves), the axis of the fiber is about perpendicular to the axis of the diffraction peaks. See Fig. 6 ▸.

**Figure 7:**
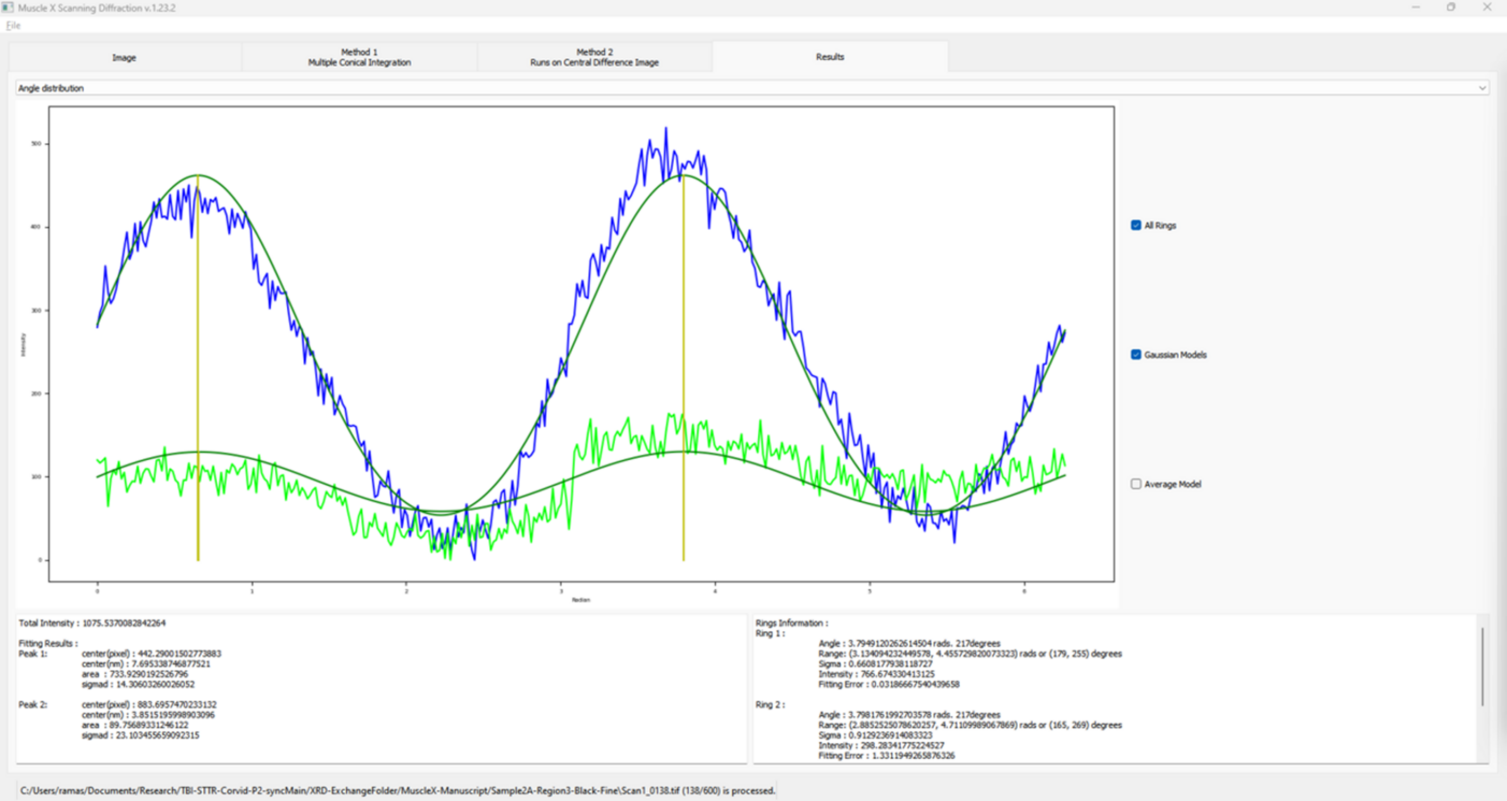
Radial integration profile and Gaussian peak fits from the raw data. The distance between peaks is ∼180° or Π radians as diffraction is centrosymmetric. The *x*-axis on this plot is the angle (radians) of the radial slice, from the origin, along which the intensities are summed, and the *y*-axis shows the summed intensity at that angle. All rings detected in the image are displayed here. The color of the display is coordinated with the ring colors in the previous integration mode.

Radial integration is performed on all diffraction patterns in a data series and multiple Gaussian curves will be fit to the 1D traces using ring locations as initial centers (Fig. 7 ▸). These Gaussian fits will be used to calculate:

(i) The distance from the center to the ring.

(ii) The standard deviation of the ring distribution in the radial direction.

(iii) The ring intensity, *i.e.* the area under the Gaussian peak fit to the raw curve.

The program will then analyze each ring to calculate the angular projection, *i.e.* the orientation of the diffraction pattern, by further fitting multiple Gaussian peaks on the 1D radial integration profile. With most fiber diffraction patterns being, at least about, centrosymmetric, the program assumes that the distance between these 1D radial integration peaks is 180° (or Π radians). These steps will then yield:

(i) The ‘orientation’ angle of the ring – axis of diffraction.

(ii) The standard deviation of the orientation angle – radial spread of the diffraction ring.

(iii) The ring intensity, *i.e.* the area under the Gaussian peak fit to the raw curve.

An angle fitting error and standard deviation (angle σ) are also calculated for each ring. Any rings with angle fitting error and angle σ ≥ 1.0 are excluded, as these correspond to isotropic diffraction rings. As mentioned previously, fibrillar assemblies produce diffraction patterns that align closely with the central axis of the fiber, whereas lamellar assemblies produce diffraction patterns perpendicular to the central axis of the fiber (Orgel *et al.*, 2019 ▸; Madhurapantula *et al.*, 2020 ▸). The display of the orientation angle for each pixel in the heatmap can be adjusted in two areas: (i) during integration in the single-image mode by checking the *Rotate 90* and *Persist Rotation* boxes, or (ii) within the heatmap interface by toggling the *Rotate 90* option. For example, the myelin sheath in the central nervous system (CNS) diffracts at a principal repeat of about 160 Å. Rings within this *d*-spacing range can therefore be used to identify myelin, and their orientations may be rotated by 90° to display the alignment of myelinated nerve fibers (or bundles) using the available toggle option.

The result of the azimuthal and radial integration processes is a series of comma separated value (CSV) files containing several metadata, settings and log files that are written into appropriately labeled subfolders in the parent folder that contains the diffraction patterns. Principally, the ‘summary.csv’ file contains the name of the image, the total intensity of selected diffraction features (rings), and number of rings detected. The ‘rings.csv’ file stores all information for all rings along with their fitting errors. Both .csv files are written into the ‘di_results’ subfolder. Additionally, there is a ‘BackgroundSummary.csv’ file that contains the average pixel value and the number of pixels outside the mask (or outside rmin if a mask is not specified) for each image processed. The calculation ignores any grid lines (detector gaps) if present. This data may later be used to scale diffraction intensities across the image series.

### 4.6. Folder mode

Upon selection of a folder containing diffraction images, the program initiates processing by retrieving all image names within the folder. A check is performed to determine whether any images remain unprocessed and are then processed individually. Subsequently, maps are generated based on the information contained in the summary.csv and rings.csv files. Processing of the images can either occur within the GUI or by invoking the program in ‘headless’ mode from the command line. This is currently only supported under linux as described in the online documentation.

Several distinct kinds of maps can be displayed in this mode, with the option of adding alignment data layers to each representation. Note that ‘best ring’ denotes the ring with the lowest fitting error of a group of user selected rings of interest (see Table 2 ▸).

**Figure 8:**
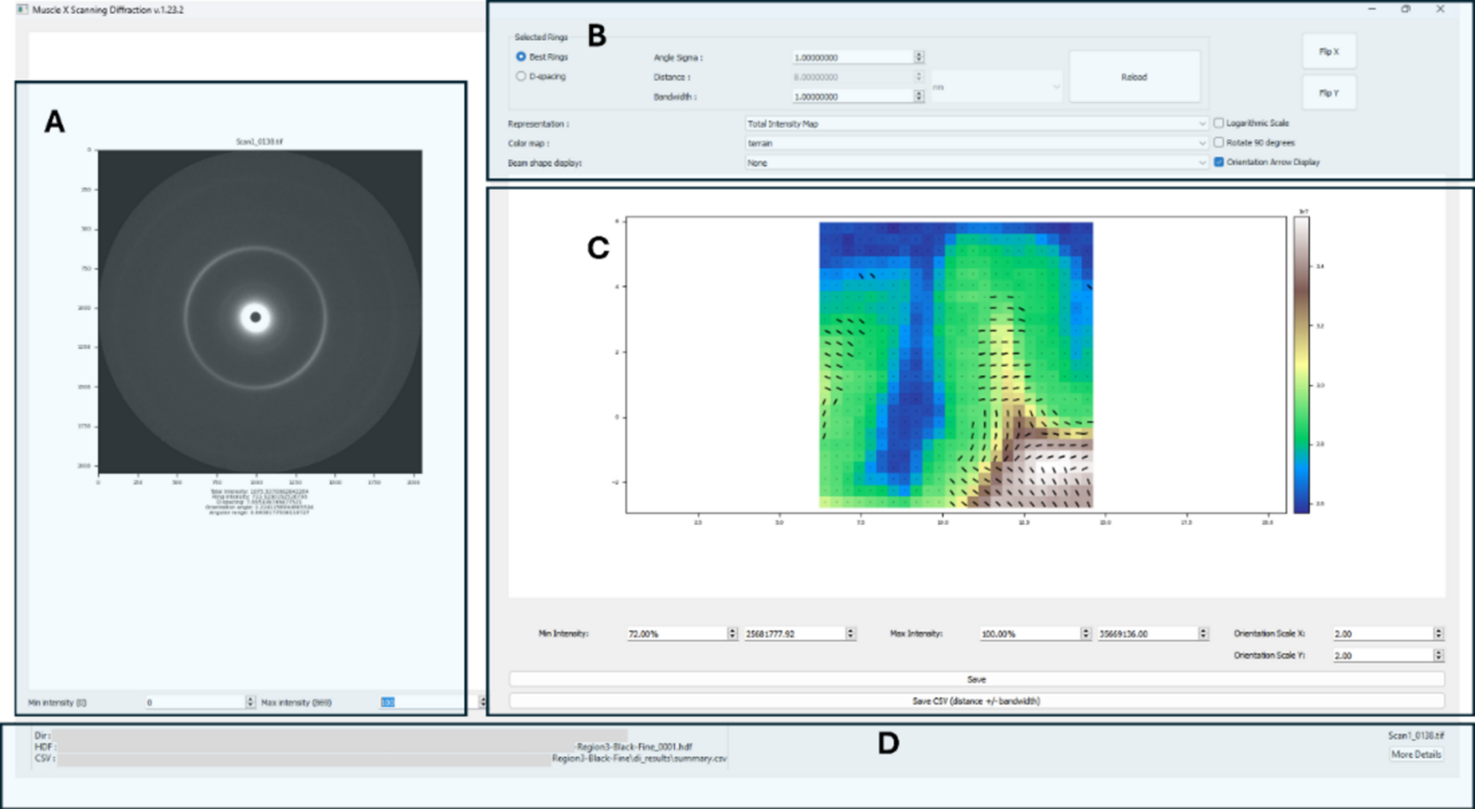
The 2D mapping interface of *MuscleX-DI*. Panel A displays the diffraction pattern that is picked from the 2D heatmap in Panel C, along with summary information such as total intensity, intensity of the ring that is being used in the heatmap, *d*-spacing of that ring, orientation angle as calculated from the ring and the angular range. Panel B is used to apply filters on rings being used to generate the 2D heatmap. Rings can be filtered based on *d*-spacing or radial distance with a fixed bandwidth. The ‘representation’ dropdown provides various heatmapping options. ‘Color map’ is used to apply colors scales to the heatmap. ‘Orientation arrow display’ overlays the fiber alignment on each pixel of the heatmap. There are more options to apply thresholds to display color on the heatmap and scale the orientation arrow that is overlayed. The user can also choose to optionally interpolate maps between data points with RBF interpolation. After all filters are applied, the heatmap can be exported as an image (png and svg supported) or a CSV file that exports the datapoints as visible on the heatmap. Panel D shows the file paths for the diffraction image and other metadata.

Each pixel within these maps corresponds to an individual diffraction image from the original image folder. To facilitate map generation, the program utilizes the setup file produced by the BioCAT scanning diffraction imaging script, if available. In the absence of a setup file, one can be generated by providing the starting and end points and *X* and *Y* step sizes of the scan. In addition to the ‘best ring’ representation, users also have the ability to filter ring display using radial distance (in pixels or nm). See Fig. 8 ▸.

### 4.7. Interpolation

Radial basis function (RBF) interpolation and techniques are available to enhance the visualization of heatmaps. RBF interpolation is utilized to reconstruct smooth and continuous parameter maps from discrete data points measured during the scanning diffraction experiments. This is useful when the beam size incident on the sample is smaller than the step size of the scan so that there are effectively gaps in information between the data points. Parameters such as total intensity, ring intensity, *d*-spacing and orientation are calculated at individual points in the scanned grid and interpolated across the entire scanned region. RBF interpolation calculates the parameter values at arbitrary points by considering their proximity to known data points. The method ensures a continuous representation of the spatial variations in the material’s structural properties. We use a multiquadric RBF kernel, and a shape parameter with a value of 1 to control the width of the basis functions. We do not employ any additional smoothing.

### 4.8. Example applications

*MuscleX-DI* was designed from the outset to provide an integrated analysis tool for scanning diffraction imaging data solution that is beamline and tissue/sample agnostic. A major application of this multi-modal imaging technique is in studying tissue mechanics at various length scales. Understanding specific tissue damage characteristics under static and dynamic load conditions at a molecular level and correlating these data at a bulk tissue level can be of great value to create better surgical techniques and design better rehabilitative regimens. For instance, research using scanning diffraction was used to characterize the muscle-tendon–leaflet transitions of a heart valve assembly under dynamic strain (Madhurapantula *et al.*, 2020 ▸). XRD scanning data were used to identify the point of ‘molecular failure’ and these data were correlated with video microscopy with fiducial markers attached to each region of the sample to track engineering strain.

**Figure 9:**
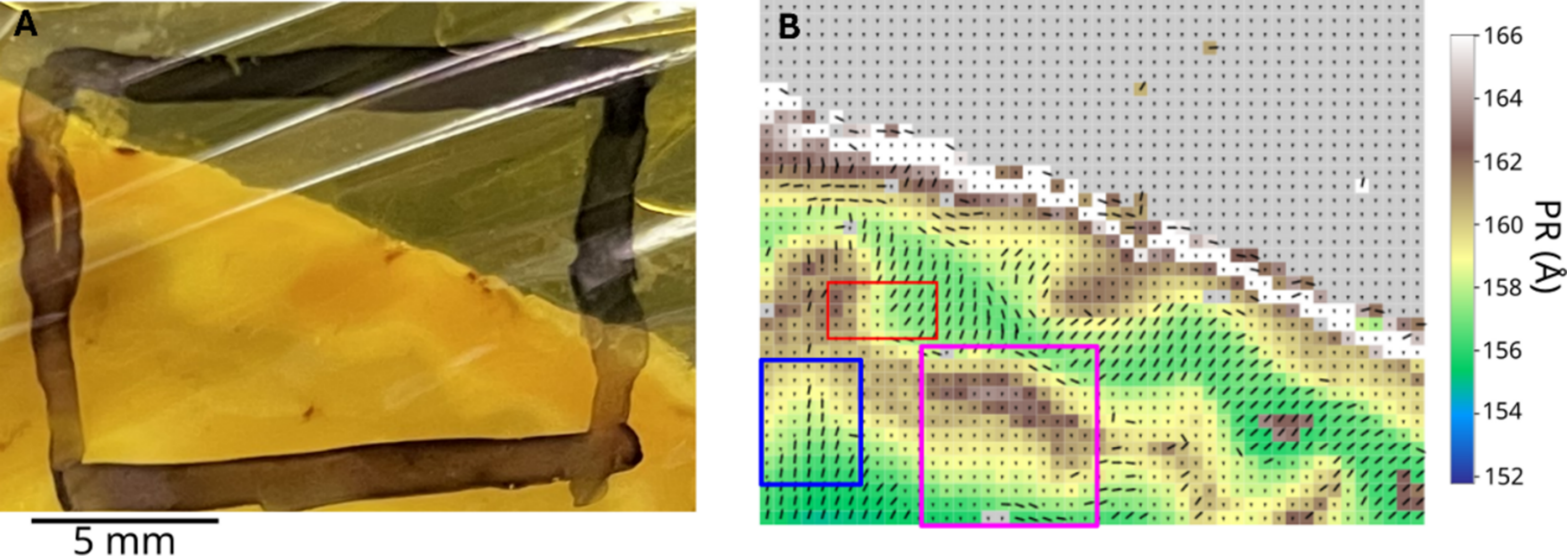
Diffraction scan of a 3 mm thick section of formaldehyde-fixed sheep’s forebrain. Panel A is a direct photograph of the section. Panel B is the 2D heatmap of myelin principal repeat with an overlay of myelin alignment at that location. The pixels without an alignment bar denote areas with isotropic diffraction patterns, *i.e.* loosely packed myelin with no preferential axis of alignment. These are notably in the visible gray matter regions of the section. Three regions of interest which demonstrate a significant transition between these tissue types are noted with different colors in panel B.

We are currently using similar approaches to understand the organization of white and gray matter and its deformation characteristics in the human brain. *MuscleX-DI* has proven invaluable in characterizing myelin organization and transitions between white and gray matter in fixed animal brain sections (Fig. 9 ▸). A 3 mm thick section of fixed sheep brain was raster scanned at a 0.3 mm step size, generating 1824 diffraction patterns. The complete analysis and rendering of these patterns were performed in about 40 minutes, revealing detailed changes in myelin packing structure based on the myelin principal repeat (PR) and alignment.

Gray matter regions exhibited isotropic diffraction with a myelin PR of 160 Å (Fig. 9 ▸). Across the gray–white matter boundary, myelin packing progressively increased, with the PR decreasing to 156 Å. The alignment of myelinated fibers also strengthened in white matter, visualized as dashes indicating fiber direction, while isotropic diffraction in gray matter is represented by dots. These findings provide significant insights into myelin organization and packing within the brain.

The deposition of extracellular matrix (ECM) around tumors is a major hurdle to developing therapeutics that penetrate this layer. New research employed the use of scanning diffraction and the insights provided by *MuscleX-DI* to observe improved drug availability in the bulk of the tumor when used with specific ECM-degrading enzymes (Rounds *et al.*, 2023 ▸; Rounds *et al.*, 2024 ▸).

There are several other areas that can readily leverage the power of this methodology and *MuscleX-DI*. The localization of specific types of amyloids can have significant implications to the pathophysiology of neurological disease such as Alzheimer’s disease (Scherpelz *et al.*, 2021 ▸; Liu & Makowski, 2022 ▸). Amyloid-laden brain tissues can be scanned using an XRD probe to determine colocalization of amyloids and specific elements in the brain (*e.g.* vasculature, ECM elements and parenchymal pathways).

### 4.9. Performance and hardware considerations

The analysis time of about 40 minutes for 1824 diffraction patterns reported in this study was performed in graphical user interface (GUI) mode on an Intel Core i7-9700K CPU (8 cores, 3.6 GHz) with 32 GB RAM running Ubuntu 22.04. Each 4 MB TIFF image was subjected to full processing, including background subtraction, *pyFAI* integration, multi-Gaussian peak fitting, orientation analysis, and two-dimensional (2D) heatmap generation using RBFinterpolation. The equivalent analysis in ‘headless’ (command-line) mode achieves comparable performance of ∼1.3 s per frame, confirming that GUI visualization contributes negligible overhead. This processing speed is appropriate for the intended use case – spatial mapping of fixed biological tissues rather than real-time beamline feedback. Typical datasets consist of a single scan per sample, with analysis conducted interactively; thus, 40 minutes of total processing time is acceptable for standard data volumes, and larger datasets can be conveniently processed overnight.

## 5. Limitations and future directions

Along with the overall *MuscleX* package, *MuscleX-DI* is currently undergoing continuous development (Jiratrakanvong *et al.*, 2024 ▸) to improve the user experience and add functionality. Current limitations that will be addressed in future releases include:

(i) Autoindexing for commonly occurring diffracting objects systems such as collagen and myelin. There is currently no detection and indexing of diffraction series based on calculated *d*-spacing. This was by design to make the software agnostic to the kind of system being analyzed so it could provide useful information on any system including those including multiple *d* spacings. Adding optional auto-indexing functionality for commonly encountered systems, however, could provide immediate insights into sample compositions and could be used to accelerate the initial setup stages described above under single image mode.

(ii) While it is possible to provide focal spot dimensions to the program, it is not currently used in the generation of heat maps, which only use the *x* and *y* step size information, which could possibly be misleading under some circumstances in terms of not representing the real resolution of the scans. If the beam size was significantly smaller than the *x* and *y* step sizes for example, this would lead to gaps between pixels. While interpolating between pixels is currently implemented, it would be better to incorporate the beam shape into the pixel representation to more accurately represent the experimental resolution.

(iii) Currently, multi-threaded processing is only possible using the linux version of *MuscleX-DI* in ‘headless’ mode. The other components of *MuscleX* have been rewritten to allow multi-threading of the GUI version of the routines so that they can run on other platforms including MS-Windows. This will be done for *MuscleX-DI* in a future release.

The computational design of *MuscleX-DI* prioritizes analytical completeness and user accessibility over raw speed. Unlike frameworks optimized for on-the-fly feedback at synchrotron frame rates, DI performs full quantitative modeling of each frame (background correction, azimuthal and radial integration, multi-Gaussian fitting, and fiber-orientation computation) to yield data ready for biological interpretation without additional scripting. For typical tissue-mapping datasets, this end-to-end automation offsets the slower per-frame throughput.

Ongoing development includes implementing multiprocessing and GPU acceleration to enhance throughput for larger datasets and near-real-time beamline feedback.

Future versions of *MuscleX-DI* will incorporate statistical and clustering functionality to facilitate multivariate analysis of complex tissue maps. Currently, users can export the per-pixel parameters (*e.g.d*-spacing, orientation, intensity) from the summary CSV files for post-processing in standard statistical environments (Python, R or MATLAB). Planned extensions include principal-component mapping and automatic phase classification.

## Figures and Tables

**Table 1:** Comparison of *MuscleX-DI* with related diffraction-analysis tools

Software	GUI	Map	Fiber metrics	Batch	Rietveld	Focus
*MuscleX-DI*	Y+CLI	Y	Built-in angle and σ	Y	N	Fiber mapping
*pyFAI*	Y	Y	API (fiber integration)	Y	N	Integration
*DAWN*	Y	Y	Sector/cake	Y	N	Workflow GUI
*Fit2D*	Y (legacy)	Partial	Wedge/sector	Batch	N	2D 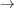 1D
*DPDAK*	Y	Y	Plugin	Y	N	SAXS/WAXS
*XRDUA*	Y	Y	N	Partial	Y	Phase maps
*GSAS-II*	Y	Indirect	Texture	Script	Y	Rietveld
*Dioptas*	Y	Batch	N	Y	N	Fast integration
*HEXRDGUI*	Y	HEDM	N	Y	Y	Grain mapping

**Table 2:** Heatmap types in folder mode

Map name	Map details
Total intensity map	Total intensity from summary.csv
*d*-space map	*d*-spacing of the best ring from the rings.csv file
Angular range maps	Standard deviation of the orientation angle (angle σ) of the best ring from rings.csv
Orientation and intensity vector field	Angle of the best ring from rings.csv as vector direction, and total intensity from summary.csv as color
Elliptical representation	Angle of the best ring from rings.csv as ellipse orientation, total intensity from summary.csv as color, and angle σ as ellipse size

## Data Availability

The *MuscleX* source code is open-source under a modified MIT license (‘the IIT license’) and is available at Github (https://github.com/biocatiit/musclex). Documentation for all routines is available at https://github.com/biocatiit/musclex/wiki. Installation packages for Microsoft Windows and MacOS are available online at https://sourceforge.net/projects/musclex/files/. It is also possible to install either the released or the current development versions of the suite using the pip or conda Python package installers.
